# Protective effects of ginsenoside Rg1 on intestinal ischemia/reperfusion injury-induced oxidative stress and apoptosis via activation of the Wnt/β-catenin pathway

**DOI:** 10.1038/srep38480

**Published:** 2016-12-02

**Authors:** Guo Zu, Jing Guo, Ningwei Che, Tingting Zhou, Xiangwen Zhang

**Affiliations:** 1 Department of Gastroenterology Surgery, The Dalian Municipal Central Hospital Affiliated of Dalian Medical University, Dalian 116033, PR China; 2 Department of Surgical Operation, Dalian Medical University, Dalian 116044, PR China; 3 Department of Neurosurgery, The Second Affiliated Hospital of Dalian Medical University, Dalian 116027, China; 4 Department of Neurology, The First Affiliated Hospital of Dalian Medical University, Dalian 116011, China

## Abstract

Ginsenoside Rg1 (Rg1) is one of the major bioactive ingredients in Panax ginseng, and it attenuates inflammation and apoptosis. The aims of our study were to explore the potential of Rg1 for the treatment of intestinal I/R injury and to determine whether the protective effects of Rg1 were exerted through the Wnt/β-catenin signaling pathway. In this study, Rg1 treatment ameliorated inflammatory factors, ROS and apoptosis that were induced by intestinal I/R injury. Cell viability was increased and cell apoptosis was decreased with Rg1 pretreatment following hypoxia/reoxygenation (H/R) in the *in vitro* study. Rg1 activated the Wnt/β-catenin signaling pathway in both the *in vivo* and *in vitro* models, and in the *in vitro* study, the activation was blocked by DKK1. Our study provides evidence that pretreatment with Rg1 significantly reduces ROS and apoptosis induced by intestinal I/R injury via activation of the Wnt/β-catenin pathway. Taken together, our results suggest that Rg1 could exert its therapeutic effects on intestinal I/R injury through the Wnt/β-catenin signaling pathway and provide a novel treatment modality for intestinal I/R injury.

Intestinal ischemia/reperfusion (I/R) injury occurs in multiple clinical settings, particularly in liver and intestine transplantation, shock and mesenteric ischemic disease^1^. Although the mechanisms of I/R injury have been extensively studied, the exact mechanisms remain to be elucidated. In the early stages of reperfusion, microcirculation fails because of endothelial cell swelling, leukocyte entrapment and vasoconstriction^2^. I/R-induced reactive oxygen species (ROS) and apoptosis cause extensive damage to intestinal epithelial cells. Moreover, the activation of inflammation induced by ROS, which produces inflammatory cytokines and oxygen-derived free radicals, could further aggravate the intestinal injury^3^,^4^,^5^.

Ginsenoside Rg1 is one of the major active and abundant ingredients in ginseng. Its chemical structure is shown in Fig. 1. Previous studies reported that Rg1 can inhibit the production of lipopolysaccharide-stimulated cytokines^6^ and protect cerebral I/R injury through attenuation of inflammation and apoptosis^7^,^8^. In a myocardial infarction rat model, Rg1 was effective at promoting angiogenesis and attenuating myocardial fibrosis, which further ameliorated ventricular dysfunction^9^. A study reported that Rg1 reduced the release of lactate dehydrogenase and intracellular ROS in a model of cardiomyocyte hypoxia-reoxygenation^10^. However, the effects of Rg1 on intestinal I/R models have not been reported.

Recent studies showed that the Wnt/β-catenin signaling pathway, which regulates multiple biological and pathological processes, including ROS, apoptosis and inflammation, is likely involved in I/R injury pathogenesis. Activation of the Wnt/β-catenin pathway protects kidneys against I/R injury by attenuating apoptosis and inflammation of tubule epithelial cells^11^. An agonist of the Wnt signaling pathway attenuated liver injury and improved the survival of rats by decreasing ROS and apoptosis induced by hepatic I/R^12^. Recent studies revealed that Rg1 activates the Wnt/β-catenin signaling pathway in neural and endothelial cells^13^,^14^.

Taken together, we propose that the protective effects of Rg1 on intestinal I/R injury involve the pro-inflammatory response, ROS generation and apoptosis *in vivo*. Pretreatment with Rg1 reduced apoptosis and inhibited ROS production, which occurred in part by activating the Wnt/β-catenin signaling pathway *in vivo* and *in vitro*. The purpose of this study was to determine the effect of Rg1 on intestinal I/R injury models and explore the potential mechanisms underlying the protective effect of Rg1.

## Results

### Histopathological changes in the intestine and survival *in vivo*

**Figure 2:**
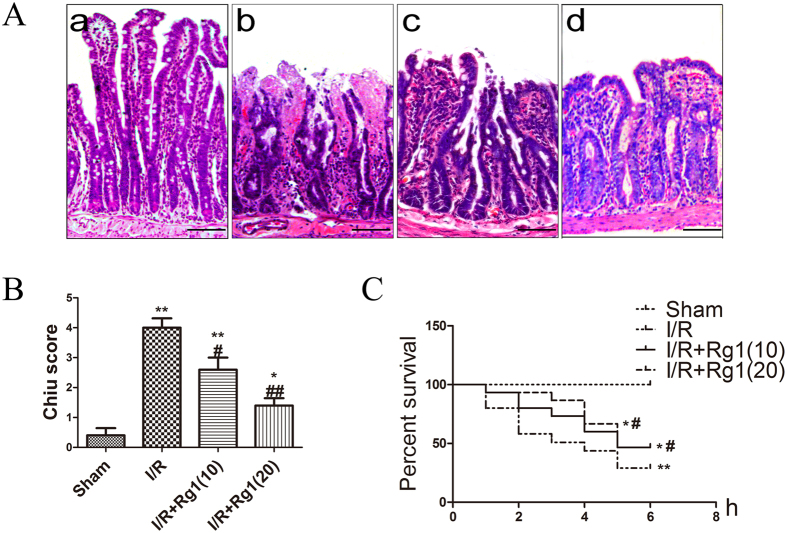
Effects of Rg1 on the macroscopic structure of intestinal tissue and survival rate after I/R injury. (**A**) Representative micrographs of intestinal tissue from (a) sham group, (b) I/R group, (c) I/R + Rg1(10) group, and (d) I/R + Rg1(20) group (bar = 50 μm). (**B**) Chiu score of the different groups (n = 5). (**C**) Survival was monitored for 6 h after I/R. Rg1 treatment reduced the mortality induced by I/R injury (n = 15). *P < 0.05 versus sham, **P < 0.01 versus sham, ^#^*P* < 0.05 versus I/R, ^##^*P* < 0.01 versus I/R.

Intestinal damage was observed in a rat model undergoing 1 h ischemia followed by 6 h reperfusion. As expected, the damage increased significantly after 6 h reperfusion compared to the sham. Rats pretreated with Rg1 were protected from I/R-induced intestinal damage, as evidenced by a significant decrease in the Chiu score (Fig. 2A,B).

Survival was assessed at 6 h after the ischemic episode in the control and treated groups (n = 15/group). As indicated in Fig. 2C, we obtained a higher rate of survival in groups treated with Rg1 compared to the I/R group.

### Rg1 alleviates the I/R-induced increase in cytokines in serum *in vivo*

**Figure 3:**
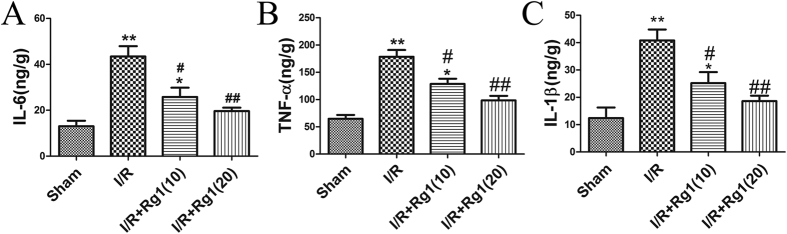
Rg1 alleviates the I/R-induced increase in cytokines in serum. (**A**) IL-6 levels of the different groups. (**B**) TNF-α of the different groups. (**C**) IL-1β of the different groups, n = 5, *P < 0.05 versus sham, **P < 0.01 versus sham, ^#^*P* < 0.05 versus I/R, ^##^*P* < 0.01 versus I/R.

Compared to the sham group, I/R-treated rats showed significantly higher expression of IL-6 (*P* = 0.0004), TNF-α (*P* = 0.0001) and IL-1β (*P* = 0.001). Pretreatment with Rg1 significantly alleviated the increase in IL-6, TNF-α and IL-1β induced by I/R compared to the I/R group rats (Fig. 3).

### Rg1 down-regulates I/R-induced intestinal MDA, SOD and GSH *in vivo*

**Figure 4:**
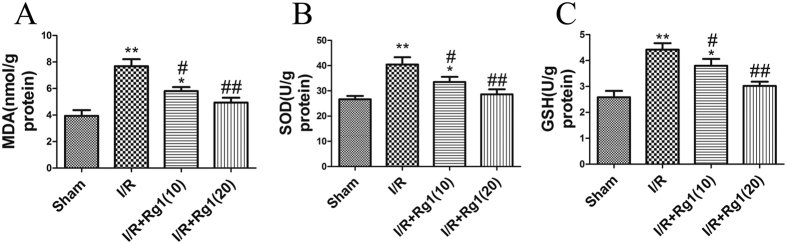
Rg1 down-regulates I/R-induced intestinal MDA, SOD and GSH. (**A**) MDA of the different groups. (**B**) SOD of the different groups. (**C**) GSH of the different groups, n = 5, *P < 0.05 versus sham, **P < 0.01 versus sham, ^#^*P* < 0.05 versus I/R, ^##^*P* < 0.01 versus I/R.

Higher intestinal tissue MDA levels (a biomarker of lipid peroxidation levels) were found in the I/R group compared to the sham group (*P* = 0.006). Rg1 administration reduced tissue MDA production. Additionally, I/R caused significant elevation of tissue SOD activity (*P* = 0.0026), and Rg1 significantly alleviated the increase in SOD induced by I/R. Tissue GSH levels were also elevated in the I/R group (*P* = 0.008), and the Rg1-administered group had reduced tissue GSH production (Fig. 4).

### Rg1 prevents I/R-induced apoptosis *in vivo*

**Figure 5:**
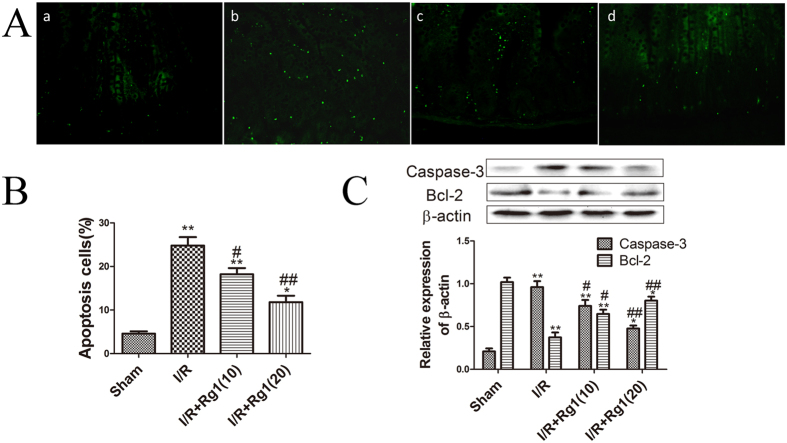
Rg1 inhibits apoptosis in intestinal tissue induced by I/R. (**A**) TUNEL staining of the different groups: (a) sham group, (b) I/R group, (c) I/R + Rg1(10) group, and (d) I/R + Rg1(20) group (×400). (**B**) Apoptotic cells of the intestine in different groups (n = 5). (**C**) The expression of apoptosis-related proteins in the different groups (n = 3). *P < 0.05 versus sham, **P < 0.01 versus sham, ^#^*P* < 0.05 versus I/R, ^##^*P* < 0.01 versus I/R.

To further examine the effects of Rg1 on I/R-induced apoptosis, we detected apoptosis in intestinal tissue using TUNEL staining and by determining the expression of apoptosis-related proteins. The results showed that I/R induced significant apoptosis (*P* = 0.0001), while Rg1 significantly decreased the number of apoptotic cells induced by I/R (Fig. 5A,B). The expression of apoptosis-related proteins showed that I/R induced intestinal apoptosis and that pretreatment with Rg1 significantly decreased the apoptosis-related proteins induced by I/R.

### Rg1 activates Wnt/β-catenin in rat intestine *in vivo*

**Figure 6:**
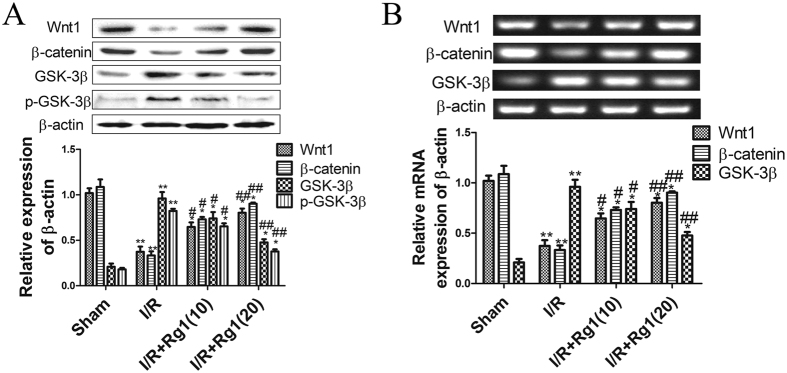
Protein and mRNAs levels of Wnt/β-catenin in rat intestinal tissues from different groups. (**A**) The protein levels of Wnt/β-catenin in rat intestinal tissues. (**B**) The mRNA levels of Wnt/β-catenin in rat intestinal tissues. The values are presented as the means ± SD (n = 3). *P < 0.05 versus sham group; ^#^*P* < 0.05 versus I/R group. **P < 0.01 versus sham group; ^##^*P* < 0.01 versus I/R group.

We observed that Rg1 can inhibit inflammatory factors and apoptosis. The Wnt/β-catenin pathway regulates the expression of a number of genes involved in inflammation and apoptosis. Therefore, we further investigated the effect of Rg1 on the Wnt/β-catenin pathway in intestinal I/R. The expression level of Wnt/β-catenin in the I/R group was significantly lower than in the sham group (Fig. 6). Rg1 significantly promoted the expression level of Wnt/β-catenin compared to the I/R rats not treated with Rg1. The results indicate that Rg1 protects against intestinal I/R injury via activation of the Wnt/β-catenin signaling pathway.

### Rg1 inhibits cell viability loss of IEC-6 cells induced by H/R injury *in vitro*

**Figure 7:**
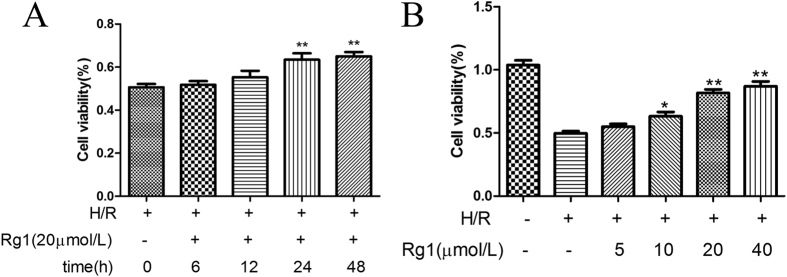
Rg1 promotes survival of IEC-6 cells under H/R conditions *in vitro*. (**A**) IEC-6 cells were exposed to H/R. The viable cells were determined using the CCK-8 assay. (**B**) IEC-6 cells were pretreated with Rg1 (5, 10, 20 and 40 μmol/L) for 24 h and then exposed to H/R, and viable cells were determined using the CCK-8 assay. All data are presented as the mean ± SD (n = 8). *P < 0.05 versus H/R group, **P < 0.01 versus H/R group.

To further explore the mechanism underlying the protective effects of Rg1, an H/R model was established using IEC-6 cells. Rg1 pretreatment effectively reduced the apoptosis induced by H/R in a dose-dependent manner. The ability of Rg1 to reverse the cell viability loss of H/R in IEC-6 cells was investigated. The measurements revealed a significant decrease in the viability of IEC-6 cells following exposure to H/R. Pre-treatment with 5 μmol/L, 10 μmol/L, 20 μmol/L or 40 μmol/L Rg1 for 24 h significantly decreased H/R-induced cell viability loss of IEC-6 cells. The results revealed that pre-treatment with 20 μmol/L Rg1 for 6, 12, 24 and 48 h showed time-dependent effects (Fig. 7A). Rg1 (5, 10, 20 or 40 μmol/L) treatment also exhibited concentration-dependent effects (Fig. 7B). Accordingly, pre-treatment with 20 μmol/L Rg1 for 24 h was selected for the following experiments because it most effectively protected IEC-6 cells from apoptosis.

### Dkk1 blocks the effects of Rg1 on the Wnt/β-catenin signaling pathway *in vitro*

**Figure 8:**
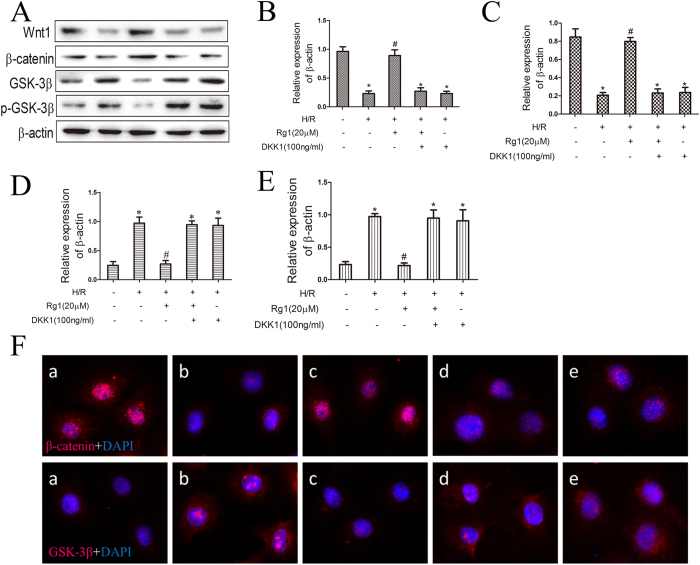
Effects of Rg1 on Wnt/β-catenin signaling following H/R, Rg1 and DKK1 treatment in IEC-6 cells. (**A–E**) Representative western blot showing levels of Wnt-1, β-catenin, GSK-3β and p-GSK-3β (n = 3). (**F**) Dual immunofluorescence staining for β-catenin (red) and DAPI (blue) or GSK-3β (red) and DAPI (blue) in IEC-6 cells. a. Control; b. H/R alone; c. H/R + 20 μmol/L Rg1; d. H/R + 20 μmol/L Rg1 + 100 ng/ml Dkk1; e. H/R + 100 ng/ml Dkk1. The data represent the mean ± SD. *P < 0.01 compared to the control group; ^#^*P* < 0.01 compared to the H/R group.

To further determine whether the Wnt/β-catenin signaling pathway is implicated in Rg1-induced protective effects *in vitro*, we examined the expression of the Wnt/β-catenin signaling pathway in IEC-6 cells. Our results indicated that the protein levels of Wnt-1 and β-catenin were significantly decreased and that GSK-3β and p-GSK-3β were significantly increased in H/R-treated cells (Fig. 8A–E). By contrast, when treated with 20 μmol/L Rg1, the protein levels of Wnt-1 and β-catenin were significantly increased and GSK-3β and p-GSK-3β were significantly decreased. The immunofluorescence results were consistent with the protein levels (Fig. 8F). These results indicate that the protective effects of Rg1 are mediated through activation of the Wnt/β-catenin signaling pathway *in vitro*.

### Dkk1 blocks the effects of Rg1 against ROS and apoptosis in IEC-6 cells induced by H/R *in vitro*

**Figure 9:**
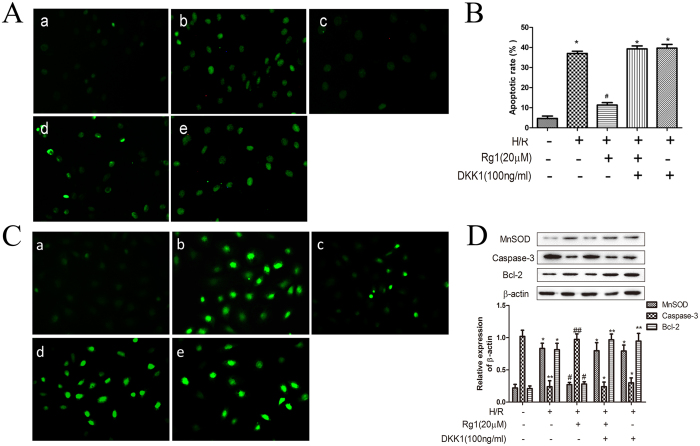
Rg1 activates the Wnt/β-catenin signaling pathway and protects IEC-6 cells against H/R-induced oxidative stress and apoptosis. (**A,B**) Apoptosis detection of IEC-6 cells using TUNEL. (**C**) ROS determination in each group. (**D**) The expression of ROS and apoptosis-related proteins in the different groups (n = 3). a. Control; b. H/R; c. H/R + 20 μmol/L Rg1; d. H/R + 20 μmol/L Rg1 + 100 ng/ml Dkk1; e. H/R + 100 ng/ml Dkk1. *P < 0.05 versus sham, **P < 0.01 versus sham, ^#^*P* < 0.05 versus I/R, ^##^*P* < 0.01 versus I/R.

To determine whether the anti-oxidant and anti-apoptotic activities of Rg1 were mediated by Wnt/β-catenin, IEC-6 cells were treated with Rg1 only, or Rg1 and DKK1 before exposure to H/R. Rg1 pretreatment diminished the ROS and apoptosis induced by H/R in IEC-6 cells. However, the anti-oxidant and anti-apoptotic effects of Rg1 were abrogated in the presence of DKK1 (Fig. 9). These results indicated that Wnt/β-catenin mediates the protective effects of Rg1 against H/R-induced oxidative stress and apoptosis.

## Discussion

I/R injury occurs when oxygen is delivered back to the tissue after a prolonged ischemia and is associated with cellular damage. The tissue damage occurs in both the ischemia and reperfusion phases^15^. Direct intestinal I/R injury may also cause distant organ dysfunction secondary to the intestinal injury^16^,^17^. Intestinal I/R injury is complex and is manifested by an inflammatory response, apoptosis and ROS production^4^. In our study, we established models of injury induced by I/R or H/R to investigate the protective effect of Rg1. We found the following: (1) I/R resulted in increased inflammatory response, ROS activity and apoptosis, and Rg1 pretreatment protected the rat intestine against I/R-induced injury; (2) Rg1 activated the Wnt/β-catenin pathway in rat intestinal I/R injury; and (3) the potential mechanisms underlying the protective effects of Rg1 were associated with different aspects of I/R injury, including suppression of ROS and decreased cell apoptosis via activation of the Wnt/β-catenin pathway.

There are multiple mechanisms underlying I/R-induced damage, and ROS and the pro-inflammatory response play important roles in its pathogenesis^18^. Evidence indicates that leukocyte adherence has an important effect with the potential to amplify some ROS production and other pro-inflammatory mediators^19^. Leukocyte activation evokes a profound vasoconstrictor effect, leading to hypoperfusion or even a no-reflow phenomenon, with complete cessation of the microcirculation^20^. Decreased ROS and pro-inflammatory response can significantly reduce injury induced by I/R^21^. Previous studies reported that Rg1 has a protective role against injury by attenuating the pro-inflammatory response and apoptosis^22^,^23^. In our study, we observed significantly higher levels of IL-1β, TNF-α, IL-6 and ROS due to intestinal I/R injury. Rg1 pretreatment inhibited these inflammatory factors and ROS. Additionally, the expression of cytokines paralleled the changes in histopathology. The present results indicate that the effects of Rg1 on alleviating I/R injury are related to its anti-inflammatory and anti-oxidative activity.

Several studies reported that under hypoxic conditions, cytoplasmic β-catenin reduces the gene transcription of other genes^24^. Shin and colleagues demonstrated that ROS-mediated cytotoxicity inhibits activation of β-catenin-dependent transcriptional activity^25^. These findings are consistent with our observations that the Wnt/β-catenin pathway is down-regulated in the rat intestine following I/R. Our results show that the protein and mRNA expression levels of Wnt/β-catenin in the intestinal I/R group were significantly decreased. Rg1 significantly promoted the expression level of Wnt/β-catenin in the intestine after I/R injury. These results indicate that Rg1 significantly activates the Wnt/β-catenin pathway in intestinal I/R injury.

The Wnt/β-catenin signaling pathway regulates the transcription of numerous target genes, including pro-apoptotic and inflammatory factors, which are implicated in I/R injury. In hepatic I/R injury, activation of the Wnt/β-catenin signaling pathway may attenuate inflammatory factors and ROS and decrease the amount of apoptosis, as well as the levels of caspase-3 activity^12^. In our study, intestinal I/R increased the number of TUNEL-positive cells *in vivo*, which was suppressed by Rg1. Consistent with the *in vivo* study, hypoxia insult followed by reoxygenation increased the number of TUNEL-positive cells, and Rg1 significantly inhibited apoptosis induced by H/R injury. Moreover, when the Wnt/β-catenin pathway was inhibited by DKK1, the suppression of Rg1 in ROS and TUNEL-positive cells was decreased. These findings suggest that ROS and apoptosis play a key role in organ damage induced by I/R and that pretreatment with Rg1 markedly reduced ROS and apoptosis induced by intestinal I/R injury via activation of the Wnt/β-catenin pathway.

Our study has several limitations. First, in this study, we investigated the protective effects of the ginsenoside Rg1 on intestinal ischemia/reperfusion injury via activation of the Wnt/β-catenin pathway; however, the exact mechanisms underlying the relationship between Rg1 and the Wnt/β-catenin pathway must be elucidated in the future. Second, our research is based on an animal model, but clinical applications are needed. Finally, Rg1, similar to ozone^26^, may trigger the healing process after intestinal I/R injury, and we may explore this in future studies.

In summary, this study demonstrated that Rg1 pretreatment protects rats from intestinal I/R injury through anti-inflammatory, anti-oxidant and anti-apoptosis effects *in vivo*. Pretreatment with Rg1 reduced apoptosis and inhibited ROS production, which occurred in part by activating the Wnt/β-catenin signaling pathway *in vivo* and *in vitro*. Because ROS and apoptosis significantly contribute to intestinal injury in I/R, we propose that Rg1 may provide a novel therapeutic strategy for the treatment of intestinal I/R injury.

## Materials and Methods

### Animals and I/R injury model

Male wild-type Sprague-Dawley rats were obtained from the Animal Center of Dalian Medical University. The study protocol and experiments were conducted according to the guidelines of the Institutional Animal Care and Use Committee of Dalian Medical University and were approved by the Institutional Ethics Committee of Dalian Medical University. The intestinal I/R injury rat model was induced as previously described^27^,^28^. Briefly, rats were fasted for 12 h with free access to tap water before surgery. After anesthetization with a pentobarbital intraperitoneal injection (40 mg/kg), rats underwent a median laparotomy and interrupted blood supply of the superior mesenteric artery (SMA) and collateral vessels with atraumatic clips. After 1 h of intestinal ischemia, the clip was removed to initiate blood reperfusion, followed by 6 h reperfusion. The sham-operated rats underwent the same procedure without vessel occlusion. At the time of sacrifice, 1-cm ileum tissue samples were collected from 5 cm proximal to the ileocecal valve.

### Administration of Rg1

Rg1 was purchased from Xiya Reagent (purity >98%, molecular formula: C_42_H_72_O_14_). A total of 60 rats were randomly divided into four groups: (1) sham group, with surgical preparation and SMA without occlusion; (2) I/R group, 1 h intestinal ischemia and 6 h reperfusion; (3) I/R + Rg1(10) group, 1 h intestinal ischemia and 6 h reperfusion with 10 mg/kg of Rg1 treatment; and (4) I/R + Rg1(20) group, 1 h intestinal ischemia and 6 h reperfusion with 20 mg/kg of Rg1 treatment.

Rg1 was dissolved in 0.9% saline and intravenously injected 1 h before surgery. The 10 mg/kg and 20 mg/kg group rats underwent surgery with left femoral vein administration of Rg1. The sham group and I/R group animals were injected with an equal volume of 0.9% saline solution. All rats were sacrificed after 6 h of reperfusion. Blood samples and intestinal tissues were obtained for analysis.

### Intestinal histologic examination

*In vivo* intestinal samples were fixed in 4% paraformaldehyde for 24 h and then dehydrated in a graded ethanol series and embedded in paraffin. Specimens (4 μm) were stained with hematoxylin-eosin (HE). Two histopathologists blinded to the group assignation independently evaluated the slides. Using the Chiu score^29^ method to evaluate intestinal mucosal damage, higher scores are interpreted as more severe damage.

### Measurement of serum interleukin-6, tumor necrosis factor-α and interleukin-1β by enzyme-linked immunosorbent assay

The *in vivo* blood samples from rats were harvested from the abdominal aorta and allowed to coagulate for 30 min at room temperature. Serum was isolated after centrifugation at 2500 rpm for 15 min. The levels of interleukin (IL)-6, serum tumor necrosis factor-α (TNF-α) and IL-1β were measured with enzyme linked immunosorbent assay kits (ENGTON bio-engineering Co, Ltd, Shanghai, China) according to the manufacturer’s protocols.

### Biochemical analysis of intestinal tissues

The *in vivo* intestinal tissues of rats were homogenized on ice in normal saline. The homogenates were centrifuged at 4000 g/min at 4 °C for 10 min. The MDA level in the supernatants was determined by measuring the thiobarbituric acid-reactive substances levels according to the manufacturer’s protocols (Nanjing Jiancheng Corp., China). The results were calculated as nmol/g protein. The SOD activity in the supernatants was evaluated according to the manufacturer’s protocols (Nanjing Jiancheng Corp., China). The results were expressed as U/g protein. Glutathione peroxidase activity was measured using the method according to the manufacturer’s protocols (Nanjing Jiancheng Corp., China). Activity was expressed as U/g protein.

### Real-time polymerase chain reaction

*In vivo* intestinal tissues of rats were collected, and the total RNA was extracted using TRIzol (Takara, Dalian, China) according to the manufacturer’s protocols. The total RNA of IEC-6 cells was isolated in the same way as described for *in vivo* experiments. First-strand cDNA was synthesized using the Reverse Transcriptase System (TransGen Biotech, Beijing, China), and target cDNAs were amplified using primer pairs for rat Wnt1, β-catenin and GSK-3β. All reverse transcription primers were synthesized by Invitrogen (Shanghai, China). The PCR primers were as follows: Wnt1 F: 5′-ATAGCCTCCTCCACGAACCT-3′, R: 5′-GGAATTGCCACTTGCACTCT-3′; β-catenin F: 5′-CGAGGACTCAATACCATTCC-3′, R: 5′-AGCCGTTTCTTGTAGTCCTG -3′; GSK-3β F: 5′-AGTGGTGAGAAGAAAGATGAGGT-3′, R: 5′-CAACTTGACATAGATCACAGGGA-3′; and β-actin F: 5′-CTGGAGAAGAGCTATGAGCTG-3′, R: 5′-AATCTCCTTCTGATCCTGTC-3′. Aliquots (5 μl) of the amplified products were loaded in 2.0% agarose gels, separated by electrophoresis, and visualized by ethidium bromide staining.

### Western blots

Intestinal tissues (*in vivo*) were lysed in RIPA buffer (Beyotime, Shanghai, China), and IEC-6 cells (*in vitro*) were also lysed in RIPA buffer. Protein concentrations were determined using a BCA Protein Assay kit (Transgen biotech, Beijing, China). Equal amounts of protein were assayed by immunoblot using Wnt1 (Santa Cruz Biotechnology, USA), β-catenin (Cell Signaling Technology, USA), GSK-3β (Cell Signaling Technology, USA), p-GSK-3β (Cell Signaling Technology, USA), cleaved caspase-3 (Cell Signaling Technology, USA) or Bcl-xL (Cell Signaling Technology, USA) or β-actin (ZSGB-bio, Beijing, China) antibodies followed by peroxidase (HRP)-conjugated goat anti-rabbit or anti-mouse IgG (ZSGB-bio, Beijing, China). Immunoreactive proteins were detected by enhanced chemiluminescence.

### Terminal deoxynucleotidyl transferase mediated dUTP nick end labeling (TUNEL) assay

TUNEL staining was performed using an *in situ* apoptosis detection kit (Roche, Branchburg, NJ) according to the manufacturer’s protocols. The *in vivo* intestinal tissues of rats were post-fixed in 4% paraformaldehyde for 2 days. Sequential 20% to 30% sucrose treatment was performed for 1 day; afterwards, the intestinal tissue samples were cryosectioned (10-μm thickness) and stained by the TUNEL method. *In vitro*, IEC-6 cells were seeded in 6-well plates and fixed with 4% PFA for 20 min. Then, the protocols were the same as for intestinal sections. Images were acquired with a fluorescence microscope (LEICA DM4000B, LEICA, Germany). Each stained section was examined, and TUNEL-positive cells were evaluated.

### Cell culture and hypoxia/reoxygenation (H/R) incubation

*In vitro* rat intestinal jejunal crypt cells (IEC-6) were obtained from the China Cell Culture Center (Shanghai, China) and were cultured at 37 °C in a 5% CO_2_ incubator. The maintenance cell media was Dulbecco’s modified Eagle’s medium (DMEM; Gibco BRL) supplemented with 10% fetal calf serum (FCS), 5 mg bovine insulin, and 50 μg/ml of penicillin/streptomycin (Beyotime, Shanghai, China). The media was changed three times per week according to standard culture protocols. The cultured cells were trypsinized with 0.25% EDTA trypsin when 90–95% confluence was achieved. To simulate *in vivo* intestinal ischemia, unless otherwise noted, cellular hypoxic conditions were created. For the hypoxic conditions, cells were incubated in a microaerophilic system (Thermo Fisher Scientific 8000, Marietta, GA, USA) at 5% CO_2_ and 1% O_2_ balanced with 94% N_2_ gas for 6 h. The cells were then cultured in normoxic conditions for reoxygenation. The IEC-6 cells were pretreated or not with 20 μmol/L Rg1 for 24 h and then treated with H/R. The 100 ng/ml DKK1 was added to IEC-6 cells 1 h prior, when needed.

### Cell proliferation assay

*In vitro* IEC-6 cell proliferation was measured indirectly using the tetrazolium salt Cell Counting Kit-8 (CCK-8, Dojindo Molecular Technologies, Inc. Tokyo, JAPAN) according to the manufacturer’s recommendations. A 96-well plate was seeded with IEC-6 cells at a total concentration of 2 × 10^3^ cells/well in 100 μL of DMEM media. Cells were allowed to attach for H/R incubation. At certain time points, cells were washed with 100 μL DMEM. Wells were incubated for 2 h with 10 μL of CCK-8, and the absorbance was measured using an ELISA microplate reader at 450 nm.

### Measurement of intracellular ROS assay after H/R

*In vitro* IEC-6 cell ROS production was detected using the fluorescent probe DCFH-DA from Molecular Probes (Invitrogen) according to the protocols described in our previous study^30^.

### Immunofluorescence

*In vitro* IEC-6 cells were post-fixed in 4% paraformaldehyde for 20 min and incubated in 0.3% Triton X-100 for 20 min. Intestinal tissue sections or cells were washed 3 times in PBS, blocked with 5% BSA solution for 1 h, and incubated with a primary antibody overnight at 4 °C. The primary antibodies were as follows: anti-rabbit β-catenin (Santa Cruz Biotechnology, Santa Cruz, CA) and anti-rabbit GSK-3β (Santa Cruz Biotechnology, Santa Cruz, CA). An Alexa Fluor 594 secondary antibody (Invitrogen Life Technologies, Carlsbad, CA, USA) was added for 1 h at 37 °C. Then, 4,6-diamidino-2- phenylindole (DAPI, Beyotime, Shanghai, China) was added as a nuclear counterstain. A Leica DM 4000B microscope or a Leica TCS SP5 microscope was used to examine staining for conventional or confocal imaging, respectively.

### Statistical analysis

In our study, the sample size calculation was based on pre-experiment data obtained from 5 animals, which were not included in the final study. We found that at least 15 subjects were required in each group to obtain a power of 90% and a two-sided α-level of 0.05 to demonstrate the protective effects of Rg1 on intestinal ischemia reperfusion.

Data are reported as the mean ± standard deviation. The Kolmogorov-Smirnov test and Shapiro-Wilk test were used to validate the assumption of normality. Statistical significance was determined using a nonparametric, 2-tailed Mann-Whitney U test for data with a non-normal distribution, including TUNEL-positive cells. Parametric unpaired two-tailed Student’s t-tests (to compare two groups) or 1-way ANOVA (for more than two groups) was used for data with a normal distribution, including western blot, RT-PCR, histological score, biochemical analysis, inflammation factors and cell proliferation activity analysis. Survival analysis at 6 h was performed using Kaplan-Meier analysis and compared using the Log-rank test. All analyses were performed with SPSS software (version 19.0; SPSS Inc, Chicago, IL). Graphs were plotted using Prism 5.0 (GraphPad Software, San Diego, CA). P < 0.05 was considered statistically significant.

## Additional Information

**How to cite this article**: Zu, G. *et al*. Protective effects of ginsenoside Rg1 on intestinal ischemia/reperfusion injury-induced oxidative stress and apoptosis via activation of the Wnt/β-catenin pathway. *Sci. Rep.* **6**, 38480; doi: 10.1038/srep38480 (2016).

**Publisher's note:** Springer Nature remains neutral with regard to jurisdictional claims in published maps and institutional affiliations.

## Figures and Tables

**Figure 1:**
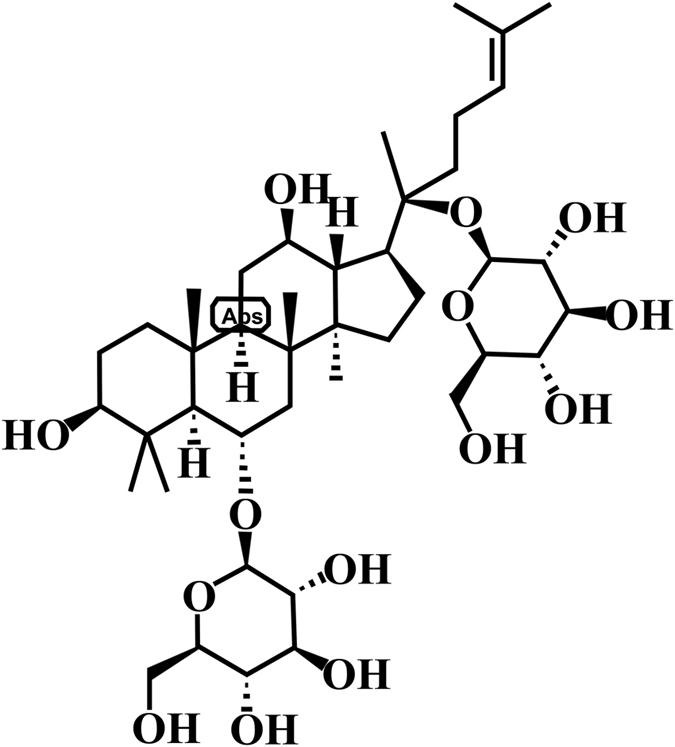
Chemical structure of Rg1.
