# Corrigendum: Protective effects of ginsenoside Rg1 on intestinal ischemia/reperfusion injury-induced oxidative stress and apoptosis via activation of the Wnt/β-catenin pathway

**DOI:** 10.1038/srep45252

**Published:** 2017-05-31

**Authors:** Guo Zu, Jing Guo, Ningwei Che, Tingting Zhou, Xiangwen Zhang, Guangzhi Wang, Anlong Ji, Xiaofeng Tian

Scientific Reports 6: Article number: 3848010.1038/srep38480; published online: 12 02 2016; updated: 05 31 2017

Guangzhi Wang, Anlong Ji and Xiaofeng Tian were omitted from the author list in the original version of this Article.

In addition, an additional affiliation for Guo Zu was omitted. The correct affiliation is listed below:

Department of General Surgery, the Second Hospital of Dalian Medical University, Dalian 116023, PR China.

As a result, the affiliation list and affiliated authors now read:

Affiliation 1

Department of General Surgery, the Second Hospital of Dalian Medical University, Dalian 116023, PR China.

Guo Zu, Guangzhi Wang, Anlong Ji & Xiaofeng Tian

Affiliation 2

Department of Gastroenterology Surgery, The Dalian Municipal Central Hospital Affiliated of Dalian Medical University, Dalian 116033, PR China.

Guo Zu & Xiangwen Zhang

Affiliation 3

Department of Operative Surgery, Dalian Medical University, Dalian 116044, PR China.

Jing Guo

Affiliation 4

Department of Neurosurgery, The Second Hospital of Dalian Medical University, Dalian 116023, China.

Ningwei Che

Affiliation 5

Department of Neurology, The First Affiliated Hospital of Dalian Medical University, Dalian 116011, China.

Tingting Zhou

The author contributions section now reads:

G.Z. designed the study. G.Z., J.G., N.C., T.Z., X.Z., G.W. and A. J. performed the experiments. G.Z., J.G., N.C. and X.Z. analyzed the data. J.G., N.C., T.Z., G.W. and A. J. contributed materials and analysis tools. G.Z. prepared the manuscript. X.T. supervised, provided lab and funding. All authors reviewed and approved the manuscript.

These errors have now been corrected in the PDF and HTML versions of the Article.

